# Association between serum lipid profile during the first and second trimester of pregnancy as well as their dynamic changes and gestational diabetes mellitus in twin pregnancies: a retrospective cohort study

**DOI:** 10.1186/s13098-023-01095-w

**Published:** 2023-06-12

**Authors:** Kexin Zhang, Wei Zheng, Xianxian Yuan, Jia Wang, Ruihua Yang, Yuru Ma, Weiling Han, Junhua Huang, Kaiwen Ma, Puyang Zhang, Lili Xu, Lirui Zhang, Xin Yan, Tengda Chen, Yujie Zhang, Guanghui Li

**Affiliations:** Division of Endocrinology and Metabolism, Department of Obstetrics, Beijing Obstetrics and Gynecology Hospital, Capital Medical University, No 251, Yaojiayuan Road, Chaoyang District, Beijing, 100026 China

**Keywords:** Twin pregnancies, Lipid profile, Gestational diabetes mellitus, Subtypes

## Abstract

**Background:**

Abnormal lipid metabolism is associated with gestational diabetes mellitus (GDM) in singleton pregnancies. Data were lacking on twin pregnancies with GDM. We explored the association between serum lipid profiles in the first and second trimesters as well as their dynamic changes and GDM in twin pregnancies.

**Methods:**

This was a retrospective cohort study of 2739 twin pregnancies that underwent a 75-g oral glucose tolerance test (OGTT) and were selected from the Beijing Birth Cohort Study from June 2013 to May 2021. Cholesterol (CHO), triglyceride (TG), high-density lipoprotein (HDL) and low-density lipoprotein (LDL) levels were measured at mean 9 and 25 weeks of gestation. We described maternal lipid levels in different tertiles that were associated with the risk of GDM stratified for age, pre-BMI, and fertilization type. GDM patients were divided into two groups according to OGTT: elevated fasting plasma glucose only (FPG group) and the rest of the GDM (non-FPG group). We estimated the relative risk of GDM with multivariable logistic regression models.

**Results:**

In this study, we found that 599 (21.9%, 599/2739) twin pregnancies developed GDM. They had increased CHO, TG, LDL, and LDL/HDL, decreased HDL levels in the first trimester, and increased TG as well as decreased HDL in the second trimester in univariate analyses, each P < 0.05. In multivariate analysis, when TG > 1.67 mmol/l (upper tertile) in elderly individuals, nonoverweight and ART groups increased the risk of GDM by 2.7-fold, 2.3-fold and 2.2-fold, respectively, compared with TG < 0.96 mmol/l (lower tertile). This effect remained in the abovementioned groups in the second trimester. Moreover, high TGs increased the risk of GDM in the FPG group (OR = 2.076, 95% CI 1.130–3.815) and non-FPG group (OR = 2.526, 95% CI 1.739–3.67) in the first trimester when TG > 1.67 mmol/l, and the rising risk in the non-FPG group as the TG tertile increased remained in the second trimester. HDL predominantly showed a negative association with elevated FPG in the second trimester (p < 0.05).

**Conclusions:**

Twin pregnancies with GDM have higher lipid levels. Increased TGs in the first and second trimesters are strongly associated with GDM, especially in elderly individuals, nonoverweight and ART groups. Lipid profiles varied among different GDM subtypes.

**Supplementary Information:**

The online version contains supplementary material available at 10.1186/s13098-023-01095-w.

## Introduction

Along with changes in maternal age, the increased use of assisted reproductive technology (ART) has resulted in a higher frequency of multiple pregnancies [1–3]. Many studies have shown that adverse perinatal outcomes in twin pregnancies are significantly more common than those in singleton pregnancies. Gestational diabetes mellitus (GDM) is one of the most common complications of adverse outcomes of pregnancy and is diagnosed as a glucose intolerance disorder first identified during pregnancy [4]. GDM is closely associated with a plethora of complications for both mothers and babies in the short and long term, such as macrosomia, preeclampsia, caesarean section [5], long-term metabolic syndrome [6] and offspring metabolic disorders [7].

The incidence of GDM in twin pregnancies is significantly higher than that in singleton pregnancies [8], at 21.9% [9] in multiple pregnancies versus 14.8% [10] in singleton pregnancies in China. Advanced maternal age, obesity, ART, and Asian ethnicity, regarded as risk factors for GDM in twin pregnancies [11–13], have been widely considered. In singleton pregnancies, lipid metabolism disorder is also associated with GDM. Lipid profiles are elevated during pregnancy and fall to normal postpartum physiologically, acting as an energy supply for foetal growth, which is induced by progesterone, oestrogen, lactogen and the accumulation of fatty acids [14, 15]. Reports show that an increase in triglycerides (TGs) and a decrease in high-density lipoprotein-cholesterol (HDL) in early pregnancy are related to GDM in singleton pregnancies [14]. Our previous study suggested that TG ≥ 1.58 mmol/L significantly increased the risk of GDM in singleton pregnancies [16]. Twin pregnancies have higher TG levels than those with singleton pregnancies throughout the whole pregnancy [17]. However, there are no data on the association between maternal lipid levels and GDM in twin pregnancies.

In addition, GDM defined by elevated fasting plasma glucose (FPG) appears to have different pathological processes from those defined by elevated postload glucose (PG). In nonpregnancy, lipid profiles vary from impaired fasting glucose (IFG) to impaired glucose tolerance (IGT) [18, 19]. A singleton pregnancy study found that circulating fatty acid levels were different in the FPG group and PG group [20]. Nevertheless, the effect of lipid profiles on different GDM subtypes is unknown.

Thus, the purpose of this study was to examine the association of lipid profiles in the first and second trimesters, as well as their dynamic changes with GDM in twin pregnancies with different ages, pre-BMIs and fertilization types. Moreover, we aimed to explore the relationship of lipid profiles in the first and second trimesters with the subsequent risk of different GDM subtypes.

## Methods

### Study subjects

**Fig. 1:**
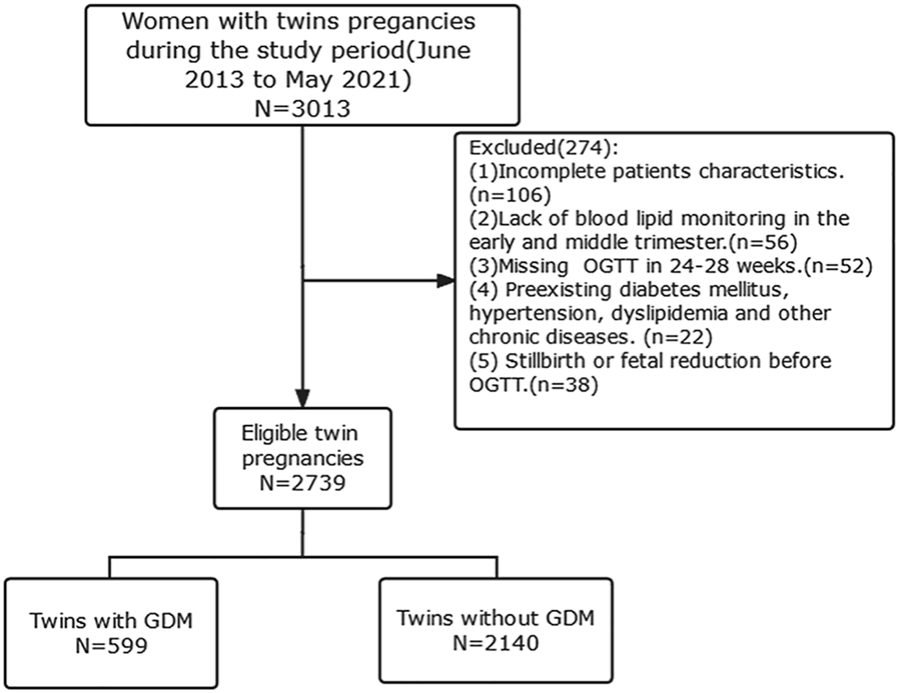
selection of study group

We conducted a retrospective cohort study of the Beijing Birth Cohort Study (ChiCTR220058395) in women aged 18–45 years with twin pregnancies who were followed up at Beijing Obstetrics and Gynaecology Hospital affiliated with Capital Medical University and delivered from June 2013 to May 2021. Data were obtained from the electronic medical records system. Exclusion criteria were as follows: (1) Incomplete maternal and infant information, (2) missing 75 g oral glucose tolerance test (OGTT) in 24–28 weeks, (3) presence of preexisting type 1 or type 2 diabetes mellitus, hypertension, dyslipidaemia, severe liver and kidney diseases, thyroid dysfunction, and other chronic diseases, and (4) stillbirth or reduction of 1 or both foetuses before OGTT. A total of 2739 pregnant women with twins met the above inclusion criteria, and their data were analysed (Fig. 1).

This study was conducted in Beijing Obstetrics and Gynaecology Hospital, Capital Medical University and was reviewed by the hospital's ethics committee (2018-ky-009-01). All procedures were performed in compliance with the Declaration of Helsinki.

### Study design

Demographic information (age, prepregnancy height and weight, GWG, number of prior pregnancies, parity, and family history) and relevant maternal and infant outcomes, as well as laboratory data, were collected from the electronic medical record system of the hospital by trained researchers. Prepregnancy body weight was self-reported. Prepregnancy body mass index (pBMI) was calculated as prepregnancy weight in kilograms divided by the square of height in metres. Moreover, we divided all participants into several groups (young group: < 35 years old; elderly group: ≥ 35 years old; nonoverweight group: < 24 kg/m^2^; overweight group: ≥ 24 kg/m^2^; ART group: natural conception group) stratified by age, pBMI and mode of conception and estimated the risk of GDM with different tertiles of lipid concentration.

Fasting plasma glucose (FPG) was detected at 7–13 weeks, and triglyceride (TG), cholesterol (CHO), high density lipoprotein-cholesterol (HDL) and low density lipoprotein-cholesterol (LDL) were measured at 7–13 weeks (9.3 weeks on average) and 24–28 weeks (25.0 weeks on average) of gestation after fasting for at least 8–12 h. FPG in early pregnancy detected by hexokinase assay and lipid panel detection were measured by the glycerophosphate oxidase method with an automatic biochemical immunoassay system (Architect ci8200; Abbott Laboratories, Chicago, IL, USA). A 75 g oral glucose tolerance test (OGTT) was performed at 24–28 weeks. Patients were instructed to fast for 8–10 h before the OGTT test and to consume a normal diet for 3 consecutive days before the test (≥ 150 g of carbohydrates a day) [20]. FPG, 1 h-PG, and 2 h-PG were determined by the glucose oxidase method with a DxC800 automatic biochemical analyser (Beckman Coulter Company, USA).

### Definition

According to the 2013 American Diabetes Association (ADA) criteria, a 75‐g oral glucose tolerance test was completed at weeks 24–28 in women not previously diagnosed with overt diabetes. GDM was diagnosed if any one of the following criteria of plasma glucose was met: fasting ≥ 5.1 mmol/L, 1 h ≥ 10.0 mmol/L, or 2 h ≥ 8.5 mmol/L. According to the OGTT results, twin pregnancies were divided into 3 groups: the non-GDM group (no elevated glucose), FPG group (isolated elevated glucose level at fasting with normal glucose levels at 1 h and 2 h), and non-FPG group (excluded GDM with isolated higher FPG which including elevated PG only and patients with both high FPG and high PG). Polycystic ovarian syndrome (PCOS) was diagnosed pre‐conception according to the modified Rotterdam Criteria, which requires the presence of 2 of the following 3 criteria: ovulation or anovulation, clinical manifestations of hyperandrogen and/or hyperandrogenemia, and ultrasound findings of polycystic ovary. Patients with other diseases, such as congenital adrenal cortical hyperplasia, Cushing’s syndrome and androgen-secreting tumours, were excluded [21]. Newborns with birth weights ≥ 4000 g delivered before this pregnancy were diagnosed with a history of macrosomia.

### Statistics

Student’s t test was used to compare continuous variables from the Twin‐GDM and Twin‐non‐ GDM groups, while the chi‐square test was used to analyse categorical variables. Repeated measurement data used repeated-measures ANOVA. Descriptive information was reported as the mean ± standard deviation for continuous variables. To examine the associations of maternal lipid concentrations in different stratifications with the risk of GDM and GDM subtypes, we calculated different tertiles of the lipid concentrations. A multivariate logistic regression was constructed based on univariate analyses, and we adjusted for age, prepregnancy BMI, family history of diabetes, ART, PCOS and FPG. Odds ratios (ORs) and 95% confidence intervals (CIs) were calculated in the multivariable logistic regression analyses. All statistical analyses were performed using SPSS version 26.0 (IBM Corp., Armonk, NY, USA). P < 0.05 was considered statistically significant.

## Results

In this study, 599 (21.9%, 599/2739) twin pregnancies developed GDM, while 2140 (78.1%, 2140/2739) had a normal oral glucose tolerance test. Clinical characteristics are described in Table 1. Compared with the non‐GDM group, GDM women had an increased maternal age, higher pre-BMI, and were more likely to have a diabetes family history, PCOS and ART (P < 0.05). They also had increased CHO, TG, LDL, and LDL/HDL levels but lower HDL levels in the first trimester (P < 0.05). In the second trimester, except for TG and HDL, other lipid profiles converged. Concordantly, ∆CHO, ∆LDL, and ∆LDL/HDL levels of women in the non-GDM group were higher than those in the GDM group, whereas there was no significant difference in TG and HDL levels between the two groups.

**Table 1:** Clinical characteristics between GDM and non-GDM in twin pregnancies

	GDM (N = 599)	Non-GDM (N = 2140)	T value/×2 value	P value
Age	34.40 ± 3.75	33.23 ± 3.92	6.516	**< 0.001**
Parity (Primipara %)	463 (77.30%)	1673 (78.18%)	0.212	0.645
Gravidity	1.60 ± 0.97	1.59 ± 0.90	0.322	0.748
Pre-pregnancy BMI	23.29 ± 3.44	22.07 ± 3.30	7.717	**< 0.001**
Family history of diabetes	69 (11.52%)	132 (6.17%)	19.707	**< 0.001**
Assisted reproductive technology	328 (63.11%)	1192 (55.70%)	10.488	**0.001**
History of PCOS	78 (13.02%)	180 (8.41%)	11.660	**0.001**
History of GDM	5 (0.83%)	9 (0.42%)	0.869	0.351
History of macrosomia delivery	4 (0.68%)	9 (0.42%)	0.243	0.872
FPG (first trimester)	4.75 ± 0.42	4.59 ± 0.37	8.462	**< 0.001**

	N = 599	N = 2140		
First trimester				
CHO	4.44 ± 0.76	4.34 ± 0.76	2.941	**0.003**
TG	1.56 ± 0.71	1.36 ± 0.68	6.129	**< 0.001**
HDL	1.60 ± 0.40	1.66 ± 0.40	-3.108	**0.002**
LDL	2.21 ± 0.61	2.10 ± 0.61	3.91	**< 0.001**
LDL/HDL	1.47 ± 0.56	1.34 ± 0.52	4.964	**< 0.001**
Test week	9.28 ± 2.03	9.31 ± 2.02	− 0.268	0.788

	N = 378	N = 970		
Second trimester				
CHO	6.20 ± 1.22	6.33 ± 1.15	− 1.765	0.078
TG	3.09 ± 1.19	2.79 ± 1.13	4.379	**< 0.001**
HDL	1.78 ± 0.41	1.88 ± 0.43	− 3.907	**< 0.001**
LDL	3.17 ± 1.06	3.28 ± 1.02	− 1.856	0.064
LDL/HDL	1.83 ± 0.62	1.82 ± 0.66	0.279	0.781
Test week	25.04 ± 2.00	24.94 ± 1.93	0.651	0.515

	N = 378	N = 970		
Changes between the first and second trimesters				
∆CHO	1.73 ± 1.05	2.01 ± 0.94	− 4.491	**< 0.001**
∆TG	1.53 ± 1.01	1.43 ± 0.91	1.697	0.09
∆HDL	0.22 ± 0.30	0.25 ± 0.31	− 1.559	0.119
∆LDL	0.86 ± 0.97	1.14 ± 0.90	− 4.896	**< 0.001**
∆LDL/HDL	0.27 ± 0.60	0.43 ± 0.56	− 4.404	**< 0.001**

Bold values represent P < 0.05

*FPG* fasting plasma glucose, *CHO* total cholesterol, *HDL* high density lipoprotein-cholesterol, *LDL* low density lipoprotein-cholesterol

We conducted two multiple logistic regression models (Tables 2 and 3), adjusted for age, prepregnancy BMI, family history of diabetes, IVF-ET, and PCOS in Model 1 and added FPG in the first trimester in Model 2. The results showed that whether we adjusted for FPG or not, higher TG levels were significantly associated with GDM in the first and second trimesters. High LDL/HDL increased the risk of GDM in Model 2 but not in Model 1 in the first trimester. Significantly, HDL showed a negative correlation with GDM in both models in the second trimester (P < 0.05).

**Table 2:**
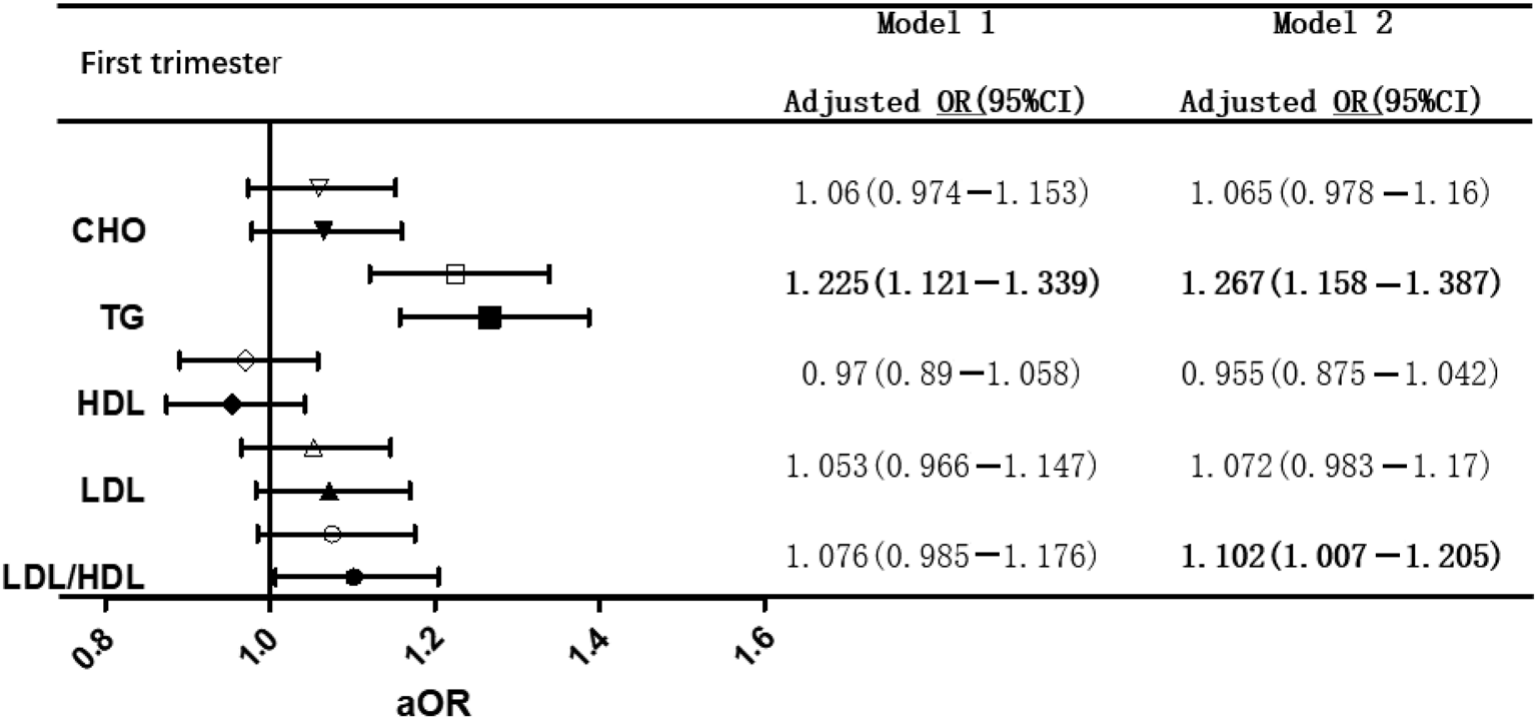
The relationship between blood lipid and GDM in first trimester of twin

Bold values represent P < 0.05

The white figure represents model 1 which adjusted age, pre-pregnancy BMI, family history of diabetes, IVF-ET, PCOS

The black figure represents model 2 which adjusted age, pre-pregnancy BMI, family history of diabetes, IVF-ET, PCOS and FPG in first trimester

*FPG* fasting plasma glucose, *CHO* total cholesterol, *HDL* high density lipoprotein-cholesterol, *LDL* low density lipoprotein-cholesterol

**Table 3:**
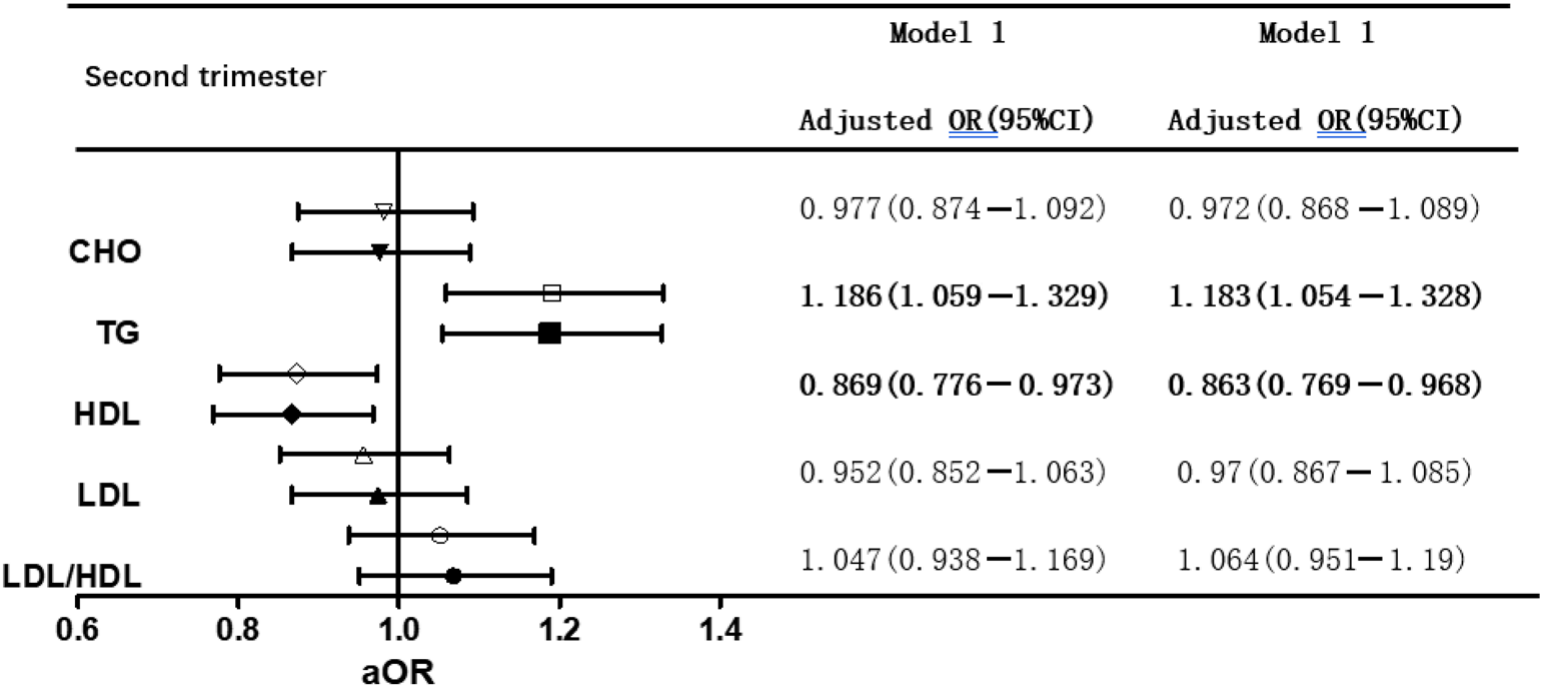
The relationship between blood lipid and GDM in second trimester of twin

Bold values represent P < 0.05

The white figure represents model 1 which adjusted age, pre-pregnancy BMI, family history of diabetes, IVF-ET, PCOS

The black figure represents model 2 which adjusted age, pre-pregnancy BMI, family history of diabetes, IVF-ET, PCOS and FPG in second trimester

*FPG* fasting plasma glucose, *CHO* total cholesterol, *HDL* high density lipoprotein-cholesterol, *LDL* low density lipoprotein-cholesterol

Moreover, we divided all participants into several groups stratified by age, pBMI and fertilization type and estimated the risk of GDM with different tertiles of lipid concentration. The results are shown in Tables 4 and 5. As TGs rise, the risk of diabetes also increases significantly in the first trimester, independent of their stratification. When TG > 1.67 mmol/l in elderly individuals, nonoverweight and ART groups, the risk of GDM increased 2.7-fold, 2.3-fold and 2.2-fold, respectively, compared with TG < 0.96 mmol/l. Nevertheless, TG remained associated in the second trimester in elderly individuals, nonoverweight and ART groups. Meanwhile, the risk of GDM increased when LDL/HDL > 1.65 mmol/l, but the correlation between HDL and GDM was nonsignificant after stratification in the second trimester Additional file 1: Tables S1, S2.

**Table 4:** Logistics regression analysis of the risk of twin pregnancies GDM based on lipid stratification in the first trimester

	Adjust OR	95%CI	P value	Adjust OR	95% CI	P value
	Age < 35			Age ≥ 35		
TG						
≤ 0.96	1			1		
0.96–1.27	1.572	1.072–2.306	**0.021**	1.435	0.901–2.287	0.128
1.27–1.67	1.452	0.975–2.162	0.066	1.929	1.208–3.081	**0.006**
> 1.67	1.804	1.188–2.74	**0.006**	2.719	1.719–4.298	**< 0.001**
LDL/HDL						
≤ 0.99	1			1		
0.99–1.30	1.117	0.765–1.632	0.566	0.814	0.526–1.262	0.358
1.30–1.65	1.157	0.789–1.696	0.456	1.138	0.743–1.743	0.553
> 1.65	1.399	0.951–2.057	0.088	0.908	0.585–1.41	0.668

	Pre-pregnancy BMI < 24			Pre-pregnancy BMI ≥ 24		
TG						
≤ 0.96	1			1		
0.96–1.27	1.63	1.157–2.296	**0.005**	1.088	0.601–1.971	0.780
1.27–1.67	1.679	1.169–2.411	**0.005**	1.348	0.768–2.368	0.299
> 1.67	2.281	1.568–3.32	**< 0.001**	1.841	1.071–3.164	**0.027**
LDL/HDL						
≤ 0.99	1			1		
0.99–1.30	0.977	0.705–1.356	0.891	0.822	0.453–1.493	0.52
1.30–1.65	1.224	0.88–1.703	0.23	0.892	0.512–1.553	0.686
> 1.65	1.156	0.811–1.649	0.423	0.975	0.578–1.643	0.923

	Assist pregnancy			Natural conception		
TG						
≤ 0.96	1			1		
0.96–1.27	1.674	1.092–2.568	**0.018**	1.344	0.882–2.047	0.169
1.27–1.67	1.728	1.131–2.64	**0.011**	1.583	1.009–2.481	**0.045**
> 1.67	2.156	1.413–3.289	**< 0.001**	2.617	1.644–4.168	**< 0.001**
LDL/HDL						
≤ 0.99	1			1		
0.99–1.30	0.889	0.61–1.294	0.539	1.067	0.686–1.662	0.772
1.30–1.65	1.094	0.757–1.579	0.633	1.232	0.79–1.92	0.357
> 1.65	1.035	0.718–1.493	0.853	1.4	0.874–2.243	0.162

Bold values represent P < 0.05

Adjusted age, pre-pregnancy BMI, family history of diabetes, IVF-ET, PCOS and FPG in first trimester

*FPG* fasting plasma glucose, *CHO* total cholesterol, *HDL* high density lipoprotein-cholesterol, *LDL* low density lipoprotein-cholesterol

**Table 5:** Logistics regression analysis of the risk of twin pregnancies GDM based on lipid stratification in the second trimester

	Adjust OR	95% CI	P value	Adjust OR	95% CI	P value
	Age < 35			Age ≥ 35		
TG						
≤ 2.12	1			1		
2.12–2.69	1.209	0.716–2.041	0.478	1.399	0.786–2.490	0.253
2.69–3.33	1.441	0.871–2.385	0.155	1.604	0.903–2.849	0.107
> 3.33	1.532	0.91–2.58	0.108	1.916	1.089–3.370	**0.024**
LDL/HDL						
≤ 1.36	1			1		
1.36–1.73	1.159	0.692–1.943	0.575	1.346	0.805–2.250	0.258
1.73–2.18	1.023	0.616–1.698	0.931	1.245	0.736–2.108	0.414
> 2.18	1.69	1.028–2.78	**0.039**	0.977	0.578–1.653	0.932

	Pre-pregnancy BMI < 24			Pre-pregnancy BMI ≥ 24		
TG						
≤ 2.12	1			1		
2.12–2.69	1.456	0.924–2.294	0.105	0.772	0.363–1.642	0.501
2.69–3.33	2.162	1.394–3.352	**0.001**	0.492	0.232–1.043	0.064
> 3.33	1.951	1.23–3.094	**0.005**	0.949	0.473–1.905	0.883
LDL/HDL						
≤ 1.36	1			1		
1.36–1.73	1.282	0.808–2.032	0.291	1.124	0.615–2.054	0.704
1.73–2.18	1.335	0.856–2.083	0.203	0.822	0.43–1.57	0.552
> 2.18	1.663	1.068–2.591	**0.024**	0.784	0.411–1.494	0.459

	Assist pregnancy			Natural conception		
TG						
≤ 2.12	1					
2.12–2.69	1.674	1.092–2.568	**0.018**	0.817	0.449–1.485	0.507
2.69–3.33	1.728	1.131–2.64	**0.011**	1.113	0.626–1.979	0.715
> 3.33	2.156	1.413–3.289	**< 0.001**	1.666	0.931–2.98	0.085

Bold values represent P < 0.05

Adjusted age, pre-pregnancy BMI, family history of diabetes, IVF-ET, PCOS and FPG in second trimester

*FPG* fasting plasma glucose, *CHO* total cholesterol, *HDL* high density lipoprotein-cholesterol, *LDL* low density lipoprotein-cholesterol

We further explored the association between blood lipids and GDM subtypes. Twin pregnancies were divided into 3 groups according to OGTT results (Table 6). Women with TG > 1.67 mmol/l experienced a 2.1-fold increased risk of GDM defined by elevated FPG only (95% CI 1.13–3.815) and a 2.5-fold increased risk of GDM in the non-FPG group (95% CI 1.739–3.67) compared with women who had concentrations < 0.96 mmol/l in the first trimester. In the second trimester, there was still a rising risk in the non-FPG group as the TG tertile grew up, but the risk was nonsignificant for the FPG group. Moreover, HDL predominantly showed a negative association with elevated FPG in the second trimester (p < 0.05). The findings showed that high LDL/HDL elevated FPG but was nonsignificant for LDL/HDL > 1.65 mmol/l. Other elevated lipid profiles had no significant effect on fasting and postload blood glucose levels.

**Table 6:** Subtypes analysis of pregnant women with twin pregnancies GDM according to OGTT results

	FPG			Non-FPG		
	Adjust OR	95% CI	P value	Adjust OR	95% CI	P value
First trimester						
CHO						
≤ 3.84	1			1		
3.84–4.29	1.157	0.635–2.105	0.634	0.905	0.61–1.343	0.621
4.29–4.80	1.204	0.662–2.188	0.543	1.03	0.62–1.711	0.908
> 4.80	1.423	0.79–2.562	0.240	0.743	0.371–1.49	0.403
TG						
≤ 0.96	1			1		
0.96–1.27	1.009	0.532–1.913	0.978	1.68	1.2–2.351	**0.003**
1.27–1.67	1.626	0.89–2.97	0.114	1.691	1.185–2.412	**0.004**
> 1.67	2.076	1.13–3.815	**0.019**	2.526	1.739–3.67	**< 0.01**
HDL						
≤ 1.35	1			1		
1.35–1.60	0.839	0.489–1.438	0.522	0.771	0.552–1.076	0.126
1.60–1.87	1.044	0.598–1.823	0.879	1.144	0.756–1.732	0.525
> 1.87	0.934	0.53–1.648	0.815	0.989	0.571–1.714	0.970
LDL						
≤ 1.71	1			1		
1.71–2.06	0.940	0.527–1.676	0.834	1.319	0.854–2.036	0.211
2.06–2.47	1.037	0.586–1.837	0.901	1.323	0.754–2.32	0.330
> 2.47	1.192	0.685–2.073	0.535	1.481	0.742–2.956	0.265
LDL/HDL						
≤ 0.99	1			1		
0.99–1.30	1.194	0.684–2.084	0.533	0.892	0.58–1.373	0.604
1.30–1.65	0.740	0.394–1.39	0.348	1.292	0.739–2.258	0.369
> 1.65	1.322	0.746–2.343	0.338	1.133	0.551–2.328	0.735
Second trimester						
CHO						
≤ 5.49	1			1		
5.49–6.21	0.780	0.382–1.594	0.496	1.523	0.885–2.621	0.129
6.21–7.02	0.888	0.448–1.763	0.735	1.618	0.75–3.49	0.220
> 7.02	0.629	0.29–1.363	0.240	2.595	0.923–7.298	0.071
TG						
≤ 2.12	1			1		
2.12–2.69	1.287	0.601–2.754	0.516	1.319	0.852–2.042	0.214
2.69–3.33	1.236	0.577–2.649	0.586	1.707	1.098–2.654	**0.018**
> 3.33	1.389	0.642–3.002	0.404	2.134	1.303–3.496	**0.003**
HDL						
≤ 1.55	1			1		
1.55–1.80	0.957	0.499–1.834	0.894	0.863	0.566–1.314	0.491
1.80–2.10	0.402	0.179–0.903	**0.027**	0.748	0.447–1.251	0.268
> 2.10	0.390	0.171–0.893	**0.026**	0.770	0.388–1.527	0.455
LDL						
≤ 2.52	1			1		
2.52–3.15	1.008	0.497–2.043	0.983	1.038	0.622–1.734	0.886
3.15–3.87	0.927	0.44–1.956	0.843	1.235	0.603–2.532	0.564
> 3.87	0.881	0.412–1.884	0.743	1.065	0.405–2.804	0.898
LDL/HDL						
≤ 1.36	1			1		
1.36–1.73	2.721	1.23–6.017	**0.013**	1.240	0.781–1.97	0.362
1.73–2.18	2.269	1.015–5.072	**0.046**	1.264	0.676–2.365	0.464
> 2.18	1.494	0.626–3.563	0.365	1.545	0.669–3.567	0.308

Bold values represent P < 0.05

Adjusted age, pre-pregnancy BMI, family history of diabetes, IVF-ET, PCOS and FPG in first and second trimester

*FPG* fasting plasma glucose, *CHO* total cholesterol, *HDL* high density lipoprotein-cholesterol, *LDL* low density lipoprotein-cholesterol

## Discussion

In this study, we found that the lipid profile of twin pregnancies with GDM differs from that of non-GDM women in the first and second trimester, but there is no significant result in their dynamic changes. Moreover, after stratification, adjusting for confounders, our results showed that twin pregnancies with higher TGs were at an increased risk of developing GDM in the first trimester in each subgroup. This effect remained in elderly, nonoverweight and ART-conceived women in second trimester, but the intensity decreased. The negative association of HDL appeared in the second trimester but disappeared after stratification. In addition, heterogeneity in the lipid profiles of GDM subtypes was shown in this study. Elevated TG levels in the first trimester increased FBG levels as well as PG levels. In the second trimester, TGs predominantly increased PG, whereas HDL showed a negative relationship with FBG.

During pregnancy, blood lipid levels increase due to progesterone and accumulation of fat to prepare for foetal growth [15, 22]. However, a large number of singleton pregnancy studies have confirmed that lipid metabolism disorders result in GDM, which manifests as high TGs in the first trimester [16, 23, 24]. In twin pregnancies, we found that elevated TGs in the first trimester also increased the risk of GDM, which is regarded as an important metabolic abnormality associated with insulin resistance [25]. Similar to our results, a Chinese study analysed 96 cases of twin pregnancies with 22 cases of GDM and found that TG levels in women with GDM were higher than that in women with non-GDM in the first and second trimesters. However, there was no significant difference in CHO levels in the whole pregnancy between GDM and non-GDM women, which was different from the results of our univariate analysis. However, in our multivariate analysis, there was no significant association between TC and GDM in the first and second trimesters of twin pregnancies [17]. A singleton pregnancy meta-analysis also found that high TGs remained consistent across each trimester in women with GDM in the second trimester [14]. However, high HDL and non-HDL results in the second trimester in women with GDM were inconsistent with our results [14] due to the heterogeneity of studies caused by age and prepregnancy BMI. Moreover, Bao et al. [22] also described different results in that high TGs and low HDL even showed a stronger association with GDM in the second trimester than in the first trimester in the multivariable model. In our study, the association between lipid profiles and GDM in the second trimester appeared to be mitigated, possibly due to the higher maternal costs for the growth of both foetuses. In addition, our study showed that there was no significant difference in the dynamic changes in lipid profiles between the GDM and control groups, which indicated that the absolute value of lipids in the first and second pregnancy are more noteworthy than their dynamic changes.

In recent years, an increasing number of studies have focused on the risk factors for GDM in twin pregnancies. Similar to our results, some studies found that twin pregnancies with GDM were more likely to be elderly [26], with a higher pre-BMI [27] and ART [28]. After we conducted further subgroup analyses stratified for maternal age, pre-BMI, and fertilization type, we found strong associations of increased TGs with the risk of developing GDM in each subgroup in the first trimester. This effect remained in elderly, nonoverweight and ART-conceived women in the second trimester. This suggests that more attention should be given to twin pregnancies with higher TG concentrations regardless of whether they have risk factors in the first trimester, as well as to specific populations in the second trimester. We previously conducted multiple regression analyses in singleton pregnancies [16] and indicated that high maternal TG levels was associated with an increased risk of GDM in the first trimester, independent of the mothers’ pre-BMI. Nevertheless, the negative relationship between HDL and GDM in the second trimester disappeared after stratification, indicating that this effect was caused by confounding factors.

In addition, our study revealed that TG increased FPG and PG levels in the first trimester. In the second trimester, TG predominantly impaired PG, while HDL showed a negative relationship with FPG. In recent years, scientists began to explore the subtypes of GDM, but most of them divided GDM into insulin sensitivity defects and insulin secretion defects, which were different from our subtypes. However, there is a connection between these two different classification methods. Powe noted that women in the GDM-sensitivity group had increased FPG and PG, whereas the GDM-secretion group only had increased PG [29]. Jill Layton found that TG increased in insulin sensitivity defects but not secretion defects in GDM [30]. These results suggested that TG elevated FPG and PG levels, probably by affecting insulin sensitivity. We can also find some evidence from nonpregnant studies. A European cohort showed that TG increased FPG and PG levels, which is consistent with our study [18]. TG predicts IFG better than other parameters [31] by impacting NO homeostasis and endothelial function to cause inflammation and oxidative stress [32]. Similar to our results, HDL also protects people with IFG from developing T2DM [18] by increasing plasma insulin and activating AMP-activated protein kinase in skeletal muscle [33]. In nonpregnancy, IFG and IGT have different pathological processes [34]. IFG patients predominantly present with hepatic IR and relatively normal muscle IR, while IGT patients present with the inverse condition [35, 36]. In our twin pregnancy study, the lipid profile also varied between the FPG and PG groups. Perhaps the pathological mechanism is similar to that of nonpregnant people.

To the best of our knowledge, this is the first study that compared lipid profiles of GDM women with twin pregnancies in the first and second trimesters as well as their dynamic changes with a large sample size. In addition, to exclude possible effects of confounding factors on GDM risk, we further analysed maternal lipid profiles associated with adjusted risks of GDM stratified for age, pre-BMI and fertilization type. Moreover, we first described the heterogeneity of maternal lipid levels in different GDM subtypes in twin pregnancies. However, our study still has some limitations. First, retrospective studies might have limitations due to potential confounding factors, such as educational status, alcohol consumption, and smoking status. Information on insulin is also insufficient, but we examined GDM subtypes according to FPG and PG levels. Second, potential selection bias may be present because our institution is a tertiary care centre. Although older and overweight twin pregnancies and those with ART are generally considered high-risk, women with uncomplicated pregnancies are less likely to visit a tertiary care centre. This may result in an increased incidence of GDM.

## Conclusion

Our study suggested that twin pregnancies with GDM have higher lipid levels. This finding implies that special attention should be given by health care providers to twin pregnancies with higher TG concentrations regardless of whether they have other risk factors. We also noted that lipid profiles varied among women with different GDM subtypes. In addition, an increasing number of new biomarkers, such as cortisol, leptin, adiponectin and human placental lactate (hPL), have been found to be related to the incidence of insulin resistance-induced diabetes [37–40]. Individualized management of dyslipidaemia in twin pregnancies in the first trimester is needed.

## Supplementary Information

**Additional file 1: Table S1.** Logistics regression analysis of the risk of twin pregnancies GDM based on lipid stratification in the first trimester. **Table S2.** Logistics regression analysis of the risk of twin pregnancies GDM based on lipid stratification in second trimester.

## Data Availability

The data is available upon reasonable request to the corresponding author.

## Abbreviations

GDM: Gestational diabetes mellitus
OGTT: 75-G Oral glucose tolerance test
CHO: Cholesterol
TG: Triglyceride
HDL: High-density lipoprotein
LDL: Low-density lipoprotein
FPG: Fasting plasma glucose
PG: Post-load glucose
IFG: Impaired fasting glucose
IGT: Impaired glucose tolerance
pBMI: Pre-pregnancy body mass index
ART: Assisted reproductive technology
ADA: American Diabetes Association
PCOS: Polycystic ovarian syndrome
ORs: Odds ratios
CI: Confidence intervals
hPL: Human placental lactogen
