# Supplementary material for: Association between serum lipid profile during the first and second trimester of pregnancy as well as their dynamic changes and gestational diabetes mellitus in twin pregnancies: a retrospective cohort study
Source: Diabetol Metab Syndr. 2023 Jun 12;15:125. doi: 10.1186/s13098-023-01095-w (PMC10259052; doi:10.1186/s13098-023-01095-w)
Supplement: Supplementary file 1 — Additional file 1: Table S1. Logistics regression analysis of the risk of twin pregnancies GDM based on lipid stratification in the first trimester. Table S2. Logistics regression analysis of the risk of twin pregnancies GDM based on lipid stratification in second trimester. [file 13098_2023_1095_MOESM1_ESM.pdf]

Supplementary table 1 Logistics regression analysis of the risk of twin pregnancies GDM based on lipid stratification in the first trimester

		Adjust OR	95%CI	P value			Adjust OR	95%CI	P value
	Age＜35					Age ≥35			
CHO	≤3.84	1			CHO	≤3.84	1		
	3.84~4.29	1.116	0.775~1.607	0.554		3.84~4.29	0.811	0.523~1.255	0.346
	4.29~4.80	1.112	0.766~1.614	0.577		4.29~4.80	1.093	0.724~1.652	0.672
	＞4.80	1.006	0.679~1.491	0.976		＞4.80	0.828	0.542~1.266	0.384
TG	≤0.96	1			TG	≤0.96	1		
	0.96~1.27	1.572	1.072~2.306	**0.021**		0.96~1.27	1.435	0.901~2.287	0.128
	1.27~1.67	1.452	0.975~2.162	0.066		1.27~1.67	1.929	1.208~3.081	**0.006**
	＞1.67	1.804	1.188~2.74	**0.006**		＞1.67	2.719	1.719~4.298	**<0.001**
HDL	≤1.35	1			HDL	≤1.35	1		
	1.35~1.60	0.642	0.448~0.919	**0.016**		1.35~1.60	0.82	0.549~1.224	0.331
	1.60~1.87	0.808	0.56~1.164	0.252		1.60~1.87	1.285	0.862~1.916	0.218
	＞1.87	0.73	0.5~1.065	0.102		＞1.87	0.943	0.619~1.437	0.785
LDL	≤1.71	1			LDL	≤1.71	1		
	1.71~2.06	1.072	0.735~1.563	0.718		1.71~2.06	1.206	0.786~1.85	0.392
	2.06~2.47	1.249	0.863~1.81	0.239		2.06~2.47	0.996	0.645~1.538	0.985
	＞2.47	1.177	0.798~1.736	0.412		＞2.47	1.071	0.702~1.633	0.751
LDL/HDL	≤0.99	1			LDL/HDL	≤0.99	1		
	0.99~1.30	1.117	0.765~1.632	0.566		0.99~1.30	0.814	0.526~1.262	0.358
	1.30~1.65	1.157	0.789~1.696	0.456		1.30~1.65	1.138	0.743~1.743	0.553
	＞1.65	1.399	0.951~2.057	0.088		＞1.65	0.908	0.585~1.41	0.668
Pre-pregnancy BMI<24					Pre-pregnancy BMI≥24				
CHO	≤3.84	1			CHO	≤3.84	1		
	3.84~4.29	0.935	0.667~1.31	0.695		3.84~4.29	1.047	0.635~1.725	0.858
	4.29~4.80	1.165	0.839~1.618	0.362		4.29~4.80	0.965	0.587~1.588	0.889
	＞4.80	0.888	0.623~1.266	0.512		＞4.80	0.95	0.583~1.546	0.835
TG	≤0.96	1			TG	≤0.96	1		
	0.96~1.27	1.63	1.157~2.296	**0.005**		0.96~1.27	1.088	0.601~1.971	0.78
	1.27~1.67	1.679	1.169~2.411	**0.005**		1.27~1.67	1.348	0.768~2.368	0.299
	＞1.67	2.281	1.568~3.32	**<0.001**		＞1.67	1.841	1.071~3.164	**0.027**
HDL	≤1.35	1			HDL	≤1.35	1		
	1.35~1.60	0.671	0.475~0.949	**0.024**		1.35~1.60	0.789	0.52~1.197	0.264
	1.60~1.87	1.015	0.73~1.41	0.931		1.60~1.87	0.921	0.577~1.471	0.73
	＞1.87	0.792	0.566~1.11	0.176		＞1.87	0.942	0.555~1.599	0.825
LDL	≤1.71	1			LDL	≤1.71	1		
	1.71~2.06	1.091	0.784~1.518	0.604		1.71~2.06	1.16	0.675~1.993	0.591
	2.06~2.47	1.11	0.796~1.547	0.538		2.06~2.47	1.071	0.624~1.837	0.804
	＞2.47	1.144	0.809~1.619	0.447		＞2.47	1.065	0.641~1.767	0.809
LDL/HDL	≤0.99	1			LDL/HDL	≤0.99	1		
	0.99~1.30	0.977	0.705~1.356	0.891		0.99~1.30	0.822	0.453~1.493	0.52
	1.30~1.65	1.224	0.88~1.703	0.23		1.30~1.65	0.892	0.512~1.553	0.686
	＞1.65	1.156	0.811~1.649	0.423		＞1.65	0.975	0.578~1.643	0.923
Assist pregnancy					Natural conception				
CHO	≤3.84	1			CHO	≤3.84	1		

	3.84~4.29	0.999	0.689~1.447	0.994		3.84~4.29	0.89	0.581~1.362	0.591
	4.29~4.80	1.026	0.713~1.477	0.891		4.29~4.80	1.196	0.787~1.816	0.402
	＞4.80	1.008	0.702~1.449	0.964		＞4.80	0.707	0.435~1.148	0.161
TG	≤0.96	1			TG	≤0.96	1		
	0.96~1.27	1.674	1.092~2.568	**0.018**		0.96~1.27	1.344	0.882~2.047	0.169
	1.27~1.67	1.728	1.131~2.64	**0.011**		1.27~1.67	1.583	1.009~2.481	**0.045**
	＞1.67	2.156	1.413~3.289	**<0.001**		＞1.67	2.617	1.644~4.168	**<0.001**
HDL	≤1.35	1			HDL	≤1.35	1		
	1.35~1.60	0.672	0.478~0.945	**0.022**		1.35~1.60	0.754	0.49~1.16	0.199
	1.60~1.87	0.919	0.655~1.288	0.623		1.60~1.87	1.063	0.687~1.647	0.783
	＞1.87	0.91	0.638~1.296	0.6		＞1.87	0.671	0.423~1.065	0.091
LDL	≤1.71	1			LDL	≤1.71	1		
	1.71~2.06	1.262	0.869~1.834	0.221		1.71~2.06	0.943	0.614~1.45	0.79
	2.06~2.47	1.173	0.808~1.701	0.402		2.06~2.47	1.063	0.692~1.634	0.781
	＞2.47	1.306	0.907~1.881	0.151		＞2.47	0.891	0.561~1.417	0.627
LDL/HDL	≤0.99	1			LDL/HDL	≤0.99	1		
	0.99~1.30	0.889	0.61~1.294	0.539		0.99~1.30	1.067	0.686~1.662	0.772
	1.30~1.65	1.094	0.757~1.579	0.633		1.30~1.65	1.232	0.79~1.92	0.357
	＞1.65	1.035	0.718~1.493	0.853		＞1.65	1.4	0.874~2.243	0.162

Adjusted age, pre-pregnancy BMI, family history of diabetes, IVF-ET, PCOS and FPG in first trimester.

Supplementary table 2 Logistics regression analysis of the risk of twin pregnancies GDM based on lipid stratification in second trimester

		Adjust OR	95%CI	P value			Adjust OR	95%CI	P value
Age ＜35					Age ≥35				
CHO	≤5.49	1			CHO	≤5.49	1		
	5.49~6.21	1.297	0.791~2.126	0.302		5.49~6.21	0.868	0.525~1.436	0.582
	6.21~7.02	0.98	0.581~1.652	0.94		6.21~7.02	0.642	0.383~1.078	0.094
	＞7.02	1.215	0.723~2.041	0.462		＞7.02	0.741	0.444~1.238	0.252
TG	≤2.12	1			TG	≤2.12	1		
	2.12~2.69	1.209	0.716~2.041	0.478		2.12~2.69	1.399	0.786~2.490	0.253
	2.69~3.33	1.441	0.871~2.385	0.155		2.69~3.33	1.604	0.903~2.849	0.107
	＞3.33	1.532	0.91~2.58	0.108		＞3.33	1.916	1.089~3.370	**0.024**
HDL	≤1.55	1			HDL	≤1.55	1		
	1.55~1.80	0.678	0.412~1.115	0.126		1.55~1.80	1.172	0.714~1.922	0.531
	1.80~2.10	0.546	0.325~0.918	**0.023**		1.80~2.10	0.968	0.559~1.677	0.908
	＞2.10	0.636	0.378~1.071	0.089		＞2.10	0.87	0.492~1.538	0.632
LDL	≤2.52	1			LDL	≤2.52	1		
	2.52~3.15	1.276	0.761~2.138	0.355		2.52~3.15	0.897	0.540~1.490	0.674
	3.15~3.87	1.177	0.703~1.969	0.535		3.15~3.87	0.892	0.512~1.555	0.687
	＞3.87	1.549	0.914~2.628	0.104		＞3.87	0.729	0.429~1.237	0.241
LDL/HDL	≤1.36	1			LDL/HDL	≤1.36	1		
	1.36~1.73	1.159	0.692~1.943	0.575		1.36~1.73	1.346	0.805~2.250	0.258
	1.73~2.18	1.023	0.616~1.698	0.931		1.73~2.18	1.245	0.736~2.108	0.414
	＞2.18	1.69	1.028~2.78	**0.039**		＞2.18	0.977	0.578~1.653	0.932
Pre-pregnancy BMI≤24					Pre-pregnancy BMI>24				
CHO	≤5.49	1			CHO	≤5.49	1		
	5.49~6.21	1.312	0.83~2.073	0.245		5.49~6.21	0.874	0.5~1.527	0.636
	6.21~7.02	1.052	0.665~1.664	0.829		6.21~7.02	0.54	0.287~1.014	0.055
	＞7.02	1.167	0.749~1.818	0.495		＞7.02	0.722	0.36~1.448	0.359
TG	≤2.12	1			TG	≤2.12	1		
	2.12~2.69	1.456	0.924~2.294	0.105		2.12~2.69	0.772	0.363~1.642	0.501
	2.69~3.33	2.162	1.394~3.352	**0.001**		2.69~3.33	0.492	0.232~1.043	0.064
	＞3.33	1.951	1.23~3.094	**0.005**		＞3.33	0.949	0.473~1.905	0.883
HDL	≤1.55	1			HDL	≤1.55	1		
	1.55~1.80	0.862	0.556~1.337	0.507		1.55~1.80	0.95	0.53~1.701	0.862
	1.80~2.10	0.684	0.434~1.079	0.102		1.80~2.10	0.637	0.318~1.274	0.202
	＞2.10	0.71	0.445~1.131	0.15		＞2.10	0.783	0.391~1.567	0.489
LDL	≤2.52	1			LDL	≤2.52	1		
	2.52~3.15	1.067	0.668~1.706	0.785		2.52~3.15	1.202	0.674~2.144	0.533
	3.15~3.87	1.324	0.833~2.104	0.236		3.15~3.87	0.643	0.331~1.249	0.193
	＞3.87	1.44	0.909~2.28	0.12		＞3.87	0.621	0.31~1.244	0.179
LDL/HDL	≤1.36	1			LDL/HDL	≤1.36	1		
	1.36~1.73	1.282	0.808~2.032	0.291		1.36~1.73	1.124	0.615~2.054	0.704
	1.73~2.18	1.335	0.856~2.083	0.203		1.73~2.18	0.822	0.43~1.57	0.552
	＞2.18	1.663	1.068~2.591	**0.024**		＞2.18	0.784	0.411~1.494	0.459
Assist pregnancy					Natural conception				
CHO	≤5.49	1			CHO	≤5.49			

	5.49~6.21	1.102	0.711~1.706	0.665		5.49~6.21	1.041	0.581~1.864	0.892
	6.21~7.02	0.683	0.434~1.074	0.099		6.21~7.02	1.117	0.606~2.061	0.722
	＞7.02	0.761	0.480~1.207	0.246		＞7.02	1.271	0.707~2.287	0.423
TG	≤2.12	1			TG	≤2.12			
	2.12~2.69	1.881	1.122~3.154	**0.017**		2.12~2.69	0.817	0.449~1.485	0.507
	2.69~3.33	2.025	1.220~3.362	**0.006**		2.69~3.33	1.113	0.626~1.979	0.715
	＞3.33	2.020	1.216~3.355	**0.007**		＞3.33	1.666	0.931~2.98	0.085
HDL	≤1.55	1			HDL	≤1.55			
	1.55~1.80	0.972	0.636~1.484	0.895		1.55~1.80	0.688	0.371~1.275	0.234
	1.80~2.10	0.771	0.482~1.234	0.279		1.80~2.10	0.596	0.321~1.104	0.1
	＞2.10	0.713	0.440~1.154	0.168		＞2.10	0.691	0.367~1.3	0.252
LDL	≤2.52	1			LDL	≤2.52			
	2.52~3.15	0.956	0.612~1.492	0.842		2.52~3.15	1.3	0.712~2.375	0.393
	3.15~3.87	0.889	0.559~1.413	0.618		3.15~3.87	1.261	0.677~2.349	0.465
	＞3.87	0.894	0.565~1.416	0.634		＞3.87	1.359	0.719~2.568	0.345
LDL/HDL	≤1.36	1			LDL/HDL	≤1.36			
	1.36~1.73	1.242	0.784~1.968	0.356		1.36~1.73	1.254	0.698~2.255	0.449
	1.73~2.18	1.032	0.659~1.618	0.890		1.73~2.18	1.247	0.676~2.302	0.480
	＞2.18	1.174	0.752~1.831	0.480		＞2.18	1.423	0.77~2.628	0.260

Adjusted age, pre-pregnancy BMI, family history of diabetes, IVF-ET, PCOS and FPG in second trimester.
